# Outcomes and Complications of the Midline Anterior Approach 3 Years after Lumbar Spine Surgery

**DOI:** 10.1155/2014/142604

**Published:** 2014-12-22

**Authors:** Charla R. Fischer, Brian Braaksma, Austin Peters, Jeffrey H. Weinreb, Matthew Nalbandian, Jeffrey M. Spivak, Anthony Petrizzo

**Affiliations:** ^1^Columbia University Medical Center, New York, NY 10032, USA; ^2^Rockford Orthopedic, Rockford, IL 61107, USA; ^3^Oregon Health and Science University, Portland, OR 97239, USA; ^4^University of Connecticut Health Center, Farmington, CT 06030, USA; ^5^New York University, Hospital for Joint Diseases, New York, NY 10003, USA

## Abstract

*Objective*. The purpose of this study was to evaluate a new questionnaire to assess outcomes related to the midline anterior lumbar approach and to identify risk factors for negative patient responses. *Methods*. A retrospective review of 58 patients who underwent anterior lumbar surgery at a single institution for either degenerative disc disease or spondylolisthesis in 2009 was performed. The outcome measures included our newly developed Anterior Lumbar Surgery Questionnaire (ALSQ), ODI, and EQ-5D. *Results*. There were 58 patients available for followup, 27 women and 31 men. The average age at surgery was 50.8 years, with an average followup of 2.92 years. The average change in ODI was 34.94 (22.7) and EQ-5D was 0.28 (0.29). The rate of complications with the anterior approach was 10.3% and there was one male patient (3.2%) with retrograde ejaculation. Determination of the effectiveness of the new ALSQ revealed that it significantly correlated to the EQ-5D and ODI (*P* < 0.05). Smoking was associated with a negative response on thirteen questions. BMP use was not associated with a negative response on any sexual function questions. *Conclusions*. Our new Anterior Lumbar Surgery Questionnaire determines patient perceived complications related to the midline anterior lumbar surgical approach.

## 1. Introduction

The anterior approach to the lumbar spine is a versatile exposure that can be utilized to access the lumbar vertebrae and disc spaces for multiple indications and is a preferred method of approach for a variety of spinal conditions and procedures [1–7]. The anterior surgical approach to the lumbar spine presents a unique set of potential complications that must be considered and discussed with the patient when selecting a surgical treatment plan for spinal disorders. These anterior approach specific complications include peritoneal perforation or damage to local nerves including the superior hypogastric plexus and the paraspinal sympathetic lumbar chain and lumbosacral plexus. Depending on the level approached, various vascular elements including the inferior vena cava, common iliac vein, ascending iliolumbar vein, low lumbar segmental vessels, and the middle sacral vessels must also be addressed to avoid vascular complications [8].

While the intraoperative surgical complications (bowel and vascular injury) related to the anterior lumbar approach have been well documented [1, 4–7, 9–15], there has been a paucity of literature related to the patient perceived postoperative difficulties and complications associated with the approach. These issues include incisional pain, change in bowel habits, and sexual dysfunction. In discussing surgical approach options, it is important to provide patients with as much information as possible to ensure that they can make an informed decision. Our new questionnaire addresses postoperative satisfaction, which may be affected by nerve damage or incision location. Patient dissatisfaction may also result from the fact that the anterior incision site and subsequent scarring are more visible to a patient compared to an analogous posterior or lateral approach. Bowel habit changes also factor into a patient's continued association with problems of the anterior surgical approach, despite surgeon determination that the incision is well healed. Sexual abnormality following the anterior approach has also received considerable attention in the literature [5, 9, 16].

The purpose of this study is to analyze the approach-related outcomes following midline anterior lumbar surgery, including patient satisfaction, patient perceived complications, and factors associated with patient perceived complications. A new newly developed Anterior Lumbar Surgery Questionnaire (ALSQ) was designed to specifically address these issues and is evaluated for correlation with previously established patient outcome measurements.

## 2. Materials and Methods

### 2.1. Study Design and Inclusion Criteria

This is a retrospective cohort analysis of adult patients who underwent anterior lumbar spine surgery in 2009. One vascular surgeon (MN) performed all of the retroperitoneal anterior lumbar approaches. After the institutional review board approval, chart information was gathered from all patients with the preoperative diagnosis of lumbar degenerative disc disease (DDD) or spondylolisthesis. Surveys were sent out to all patients as well as phone calls and 58 were available for followup. All male and female patients of age 18–80 years with lumbar DDD or spondylolisthesis treated with anterior lumbar fusion were included. Exclusion criterion was patients with prior spinal surgery at the same vertebral levels, trauma, ankylosing spondylitis, rheumatoid arthritis or chronic autoimmune conditions, idiopathic scoliosis, neoplasm, or preoperative spinal infection.

### 2.2. Data Collection

The demographic characteristics included age, gender, BMI, smoking status, worker's compensation status, prior surgery status, postoperative complications, and revisions. Intraoperative information collected included interbody device type (allograft, metal, and PEEK), bone morphogenetic protein use, number of fusion levels, specific fusion levels, if a posterior procedure was performed, if the posterior procedure was open or percutaneous, and intraoperative complications. A Pfannenstiel incision was performed for all isolated L5-S1 procedures as well as a vertical midline incision for all other procedures. Thus, data about incision type is included with the specific fusion levels information. The medical records were reviewed for instances of postoperative complications prior to discharge and in the follow-up period.

### 2.3. Questionnaires

The newly developed Anterior Lumbar Surgery Questionnaire (ALSQ), shown in Appendix A, includes questions related to known specific complications after anterior lumbar surgery [6, 10, 12]. These questions are designed to ascertain patient perceived outcomes specifically related to pain, appearance, and functional impairment of the anterior incision, as well as changes in bowel and bladder habits, and sexual function. Patients also filled out ODI and EuroQol questionnaires and the answers on these previously validated surveys were matched to specific questions on our new questionnaire to evaluate for an agreement [2, 5, 6, 13, 17, 18]. Patient demographic and intraoperative data were evaluated for significant association with patient perceived complications.

### 2.4. Statistical Analysis

The data were analyzed using SPSS version 17 software (Chicago, IL). Statistical analysis was performed in the following manner. For most variables for which data were collected preoperatively and postoperatively, paired *t*-tests were used to determine whether there was a significant change between time-points. Unpaired Student *t*-test was used to assess the difference of continuous measures across groups. Chi-square test was used for dichotomous data analysis. ANOVA test was used to compare more than 2 categories across multiple variables. A *P* value of <0.05 was considered statistically significant.

## 3. Results

### 3.1. Demographics

There were 58 patients, 27 women and 31 men, with an average age at surgery of 49.2 years (±11.9 years). The average followup postoperatively was 2.92 years (±0.29 years). There were 43 patients with a diagnosis of lumbar degenerative disc disease, 15 patients with lumbar spondylolisthesis. Intraoperative complications related to the anterior approach include four vascular complications, 6.25%. Postoperative complications related to the anterior approach occurred at 1.7% and included an incisional hernia leading to an abdominal hernia repair. While there were no complaints of retrograde ejaculation noted in the extensive chart review, the ALSQ results demonstrated one male patient with this complication, with a rate of 3.2% (1/31 men). The preoperative and intraoperative patient characteristics are shown in Tables 1 and 2.

### 3.2. HRQOL Outcomes

Of the 58 total patients available for followup, 23 had preoperative EuroQol and ODI values, 14 men and 9 women. In this group, the average preoperative ODI was 58.78 and the average preoperative EuroQol was 0.41. All patients sustained a statistically significant improvement in both ODI and EuroQol as shown by an average 3-year change in ODI which was 34.9 (±22.7) and average EQ-5D change was 0.28 (±0.29). The improvements in HRQOL measures are shown in Table 3.

### 3.3. Questionnaire Results

The entire questionnaire with percentage of responses is shown in Appendix A. Overall, 35% of patients responded yes to having pain in the region of their anterior incision, 20% rate it as a 3 out of 10, 45% of the patients experience the pain every day, 30% state that it is improving since the first year after the surgery, and 43% deny sensitivity or numbness. With respect to appearance, 70% deny any bulging or asymmetry, 31% rate their satisfaction as 10 out of 10, 66% state that they see no change in the appearance of their incision, and 84% have not had any further treatment of their incision.

In relation to patient function, 19% of patients state that the anterior incision limits their daily functions. Eighty-one percent of patients stated that their bowel function is the same as prior to surgery. Of the patients who stated that their bowel function had changed, 72% stated that their main complaint was constipation. When asked about the frequency of their bowel complaints, only 8% of patients describe it as daily. Thirteen percent of patients have sought advice from a gastroenterologist for their new bowel complaints.

For urinary issues, 22% of patients state that their urinary habits are worse after surgery and 14% have sought the advice of urologist. For changes in sexual function after surgery, the questions were divided into gender specific issues, but combined for statistical analysis. In general, 48% of patients have an unchanged sex life postoperatively, 50% of men have no difficulty in achieving an erection, and 78% of women have no difficulty with genital moistness. One male patient reported having no ejaculation with orgasm after surgery, which meets the criteria of retrograde ejaculation.

When comparing the responses on the ALSQ to the postoperative EQ-5D and ODI responses for all 64 patients, we found that the responses were significantly similar. We paired specific questions on the ALSQ to specific questions on both EQ-5D and ODI and evaluated similar responses across all three questionnaires. There is a significant association with all of the questions, and the results are shown in Table 4. This demonstrates that the questions asked in the anterior lumbar survey are correlated with established and validated questions on both EQ-5D and ODI.

### 3.4. Risk Factors for Worse Patient Survey Responses

The ALSQ responses were evaluated for association with demographic, intraoperative, and postoperative factors. The statistical significance level for association with complications was set to *P* = 0.20 since this is the recommendation by CONSORT, the international committee for medical trials, when evaluating adverse events [19].

The risk factor of age older than 50 years old was associated with a worse response on 1 question and a better response on three questions. Patients older than 50 years old are more likely than patients younger than 50 years old to answer yes to anterior incision pain (90% versus 58% resp., *P* = 0.10) and less likely to complain of sensitivity or numbness at the incision (45% versus 68%, *P* = 0.06) to complain of worse bowel function after surgery (6.25% versus 22%, *P* = 0.07) or to seek a medical evaluation for bowel complaints (2.9% versus 24%, *P* = 0.01).

Male gender, as a risk factor, was associated with having a worse response than women on seven questions on the survey. Men are more likely to complain of anterior incision pain “everyday” or “frequently” (88% versus 61%, *P* = 0.16), complain of bulging or asymmetry at the anterior scar (40% versus 21%, *P* = 0.11), have worsening bowel function after surgery (21% versus 10%, *P* = 0.12), complain of urinary issues “everyday” or “frequently,” (27% versus 13%, *P* = 0.14), seek medical attention for new urinary issues after surgery (21% versus 9%, *P* = 0.20), complain of difficulty with orgasm (46% versus 17%, *P* = 0.02), and more likely to complain of changes in genital sensation (32% versus 17%, *P* = 0.19).

The risk factor of BMI greater than 30 was associated with a worse response compared to patients with a BMI less than 30 on three questions. Patients with a BMI > 30 are more likely to complain of pain at their anterior incision (30% versus 13%, *P* = 0.08), worsening bowel function after surgery (22% versus 8%, *P* = 0.11), and complain of difficulty with bowel habits “everyday” or “frequently” (36% versus 8%, *P* = 0.01).

Smoking is the greatest risk factor for poorer outcome. It was associated with worse responses than nonsmokers or prior smokers on thirteen questions. On the questions related to pain and appearance, smokers are more likely to complain of anterior incision pain (56% versus 30%, *P* = 0.13), have anterior incision pain “everyday” or “frequently” (100% versus 64%, *P* = 0.12), feel that their anterior scar pain is “getting worse” since surgery (33% versus 6%, *P* = 0.01), admit to sensitivity or numbness at the anterior scar (89% versus 51%, *P* = 0.03), and have undergone further treatment for their anterior scar/incision (44% versus 9%, *P* = 0.01). In terms of functional questions, smoking patients complain that the anterior incisional pain limits activities (55% versus 13%, *P* = 0.003), feel that their bowel function is getting worse since surgery (50% versus 8%, *P* = 0.001), complain of difficulty with bowel habits “everyday” or “frequently” (44% versus 13%, *P* = 0.02), and have undergone a new medical evaluation for changes in bowel function (33% versus 9%, *P* = 0.05). On the sexual function questions, smokers are more likely to admit to a less than normal sex life after surgery (77% versus 47%, *P* = 0.09), have difficulty with maintaining an erection in men (100% versus 50%, *P* = 0.06), have difficulty with orgasm (57% versus 27%, *P* = 0.11), and changes in genital sensation (50% versus 20% *P* = 0.07).

The risk factor of a worker's compensation claim was associated with a worse response on eight questions and a better response on one question compared to nonworker's compensation patients. On the pain and appearance questions, worker's compensation patients are more likely to have numbness or sensitivity at the anterior scar (78% versus 53%, *P* = 0.17), have bulging or asymmetry at the anterior scar (50% versus 27%, *P* = 0.14), and seek further treatment for their anterior scar (40% versus 11%, *P* = 0.02). On the function questions, worker's compensation patients are more likely to seek a medical evaluation for bowel changes (30% versus 9%, *P* = 0.07), seek a medical evaluation for urinary issues (30% versus 11%, *P* = 0.13), and more likely to have a less than normal sex life (75% versus 48%, *P* = 0.16), difficulty with maintaining an erection in male patients (77% versus 47%, *P* = 0.13), and changes in genital sensation (56% versus 26%, *P* = 0.08). Worker's compensation patients are less likely to complain of constipation (33% versus 82%, *P* = 0.01).

Degenerative disc disease, as a risk factor, was associated with a worse response than spondylolisthesis patients on seven questions. On the pain and functions questions, patients with degenerative disc disease are more likely to have pain at their anterior scar (38% versus 19%, *P* = 0.17), complain of worsening urinary function (28% versus 6%, *P* = 0.08), have urinary symptoms “everyday” and “frequently” (24% versus 6.67%, *P* = 0.15), and seek a medical evaluation for urinary symptoms (19% versus 0%, *P* = 0.06). On the sexual function questions, patients with degenerative disc disease patients have a less than normal sex life (59% versus 29%, *P* = 0.05), have difficulty with orgasm (33% versus 7%, *P* = 0.03), have a less than normal ejaculate (33% versus 7%, *P* = 0.06), and complain of changes in genital sensation (31% versus 7%, *P* = 0.06).

Bone morphogenetic protein use as a risk factor, compared to patients in whom BMP was not used, was associated with a worse response on one question and a better response on five questions. Patients in whom BMP was used are more likely to complain of urinary incontinence as their urinary complaint when they complained of worsening urinary function (40% versus 8%, *P* = 0.10). Better responses for patients with BMP use included less likelihood of worsening urinary function after surgery (14% versus 32%, *P* = 0.09), less likely to complain of urinary issues “everyday” or “frequently” (12% versus 30%, *P* = 0.08), less likely to complain of difficulty with orgasm (17% versus 46%, *P* = 0.02), less likely to have less ejaculate (16% versus 40%, *P* = 0.05), and less likely to have changes in genital sensation (13% versus 40%, *P* = 0.02).

The different interbody devices, as a risk factor for worse patient outcomes, were associated with significantly different patient response on only three questions. Patient in whom FRA was used had a significantly higher rate of poor sexual function (67%, compared to 45% in PEEK, 25% in metal, and 33% in TDR, *P* = 0.14) and higher rate of difficulty with orgasm (44%, compared to 19% in PEEK, 11% in metal, and 33% in TDR, *P* = 0.17). Patients with metal implants had a significantly lower rate of changes in genital sensation (0% compared to 34% in FRA, 18% in PEEK, and 33% in TDR, *P* = 0.19).

Evaluation of number of fusions levels found that 2-fusion levels were associated with worse responses than 1-fusion level on four questions. Two-level fusion patients had a higher rate of diarrhea after surgery (29% compared to 17%, *P* = 0.19), visiting a GI specialist at (23%, compared 6%, *P* = 0.19) a higher rate of difficulty marinating an erection (75% compared to 40%, *P* = 0.17), as well as an increased change in genital sensation (39% compared to 17%, *P* = 0.12). Patients with a two-level fusion had a better response on one question. Patients with two fusion levels, 44% rated the pain frequency as rarely compared to 9% of patients with single level fusions, *P* = 0.18.

When evaluating the specific fusion levels, there was association with worse outcomes on 4 questions. Fusions at L3/4 or L3-L5 level were associated with both sensitivity and numbness at the incision, as well as bulging or asymmetry (75%, *P* = 0.10, and 50%, *P* = 0.10, resp.). Fusions of L4/5 or L4-S1 were associated with difficulty in achieving an erection and change in genital sensation (70%, *P* = 0.06, 42%, *P* = 0.05, resp.).

A comparison of anterior only and anterior-posterior surgeries was performed. Anterior only surgeries were associated with higher rate of answering improving abdominal shape after surgery. (80% versus 9%, *P* = 0.011). Additionally, patients who underwent an anterior-posterior approach demonstrated a higher rate of difficulty maintaining an erection, (65% versus 25%, *P* = 0.15).

Of the patients who underwent posterior procedures, a comparison of open and percutaneous approaches was performed and a difference was found on 4 questions. Open posterior procedures had a higher rate of bulging or asymmetry of the anterior incision (39% versus 17%, *P* = 0.13) but also a higher rate of no change to the abdominal shape (73% versus 36%, *P* = 0.07). Patients who underwent percutaneous procedures had a higher rate of no change to their bowel function (86%, versus 64%, *P* = 0.14) but a lower rate of answering no change to sexual function (37% versus 65%, *P* = 0.135).

The risk factor of complications was associated with a worse response than patients without complications on two questions. Patients who had complications are more likely to seek a medical evaluation for changes in bowel habits after surgery (33% versus 11%, *P* = 0.11) and more likely to have problems achieving an erection/moistness after surgery (100% versus 42%, *P* = 0.12).

Prior surgery status was associated with a worse response than primary surgery patients on five questions. Patients who had undergone a prior surgery are more likely to have pain at the anterior incision (45% versus 28%, *P* = 0.18), complain of sensitivity or numbness at the anterior scar (70% versus 50%, *P* = 0.14), and have bulging or asymmetry at the anterior scar (50% versus 21%, *P* = 0.02), problems achieving erection/moistness after surgery (83% versus 36%, *P* = 0.04), and male patients complained of difficulty maintaining an erection (83% versus 50%, *P* = 0.14).

## 4. Discussion

Outcomes from this study are similar to those in the literature for the anterior approach and demonstrate that overall patients do well after anterior lumbar surgery with an average 3-year change in ODI of 34.3 and average EQ-5D change of 0.29. The overall anterior approach related complications rate was 10.7%. The complication rate of vascular injury in this study was 6.2%, which is similar to 7% described by Rajaraman et al. in 1999 [14]. In their study, evaluating for visceral and vascular injury after anterior lumbar surgery, the total rate of anterior approach related complications was 38.3%, significantly higher than our rate of 10.7%.

Retrograde ejaculation occurs when the bladder sphincter is unable to prevent semen from entering the urinary bladder during ejaculation. Our rate of retrograde ejaculation of 2.9% is substantially lower than previously described rates in the literature, 4.1–11.6% in the total patient population [14, 20]. In the study by Rajaraman et al., the retrograde ejaculation rate was 9.6%, and they recovered by 15 months after surgery. Carragee et al. have cited the significant increase in retrograde ejaculation in anterior lumbar surgical cases where recombinant BMP-2 (Infuse, Medtronic), a commercially available growth factor used in spinal fusion, was utilized [5, 9, 16]. Burkus et al. have recently evaluated 5 prospective trials and found an association between BMP use and the transperitoneal approach, but this was not significant [21]. However, in the cohort involved in the present study, only one instance of retrograde ejaculation was reported despite BMP use in 55% of the patients. Additionally, no association between BMP use and sexual dysfunction was found. These findings are in contrast to the recent studies citing an association between BMP and RE [5, 9, 16, 21].

The results of this study demonstrate that this questionnaire is useful in assessing patient reported outcomes specific to the midline anterior lumbar approach. The correlation of the specific question on the ALSQ to the corresponding questions on the ODI and EQ-5D demonstrate that we adapted the concepts for the anterior midline approach. The EQ-5D, also known as the EuroQol, is a good general health survey used commonly in European registries due to its brevity [22–24]. Another good general health questionnaire would be the SF-36, developed and validated in the United States but often not completely filled out by patients. The ODI, or Oswestry Disability Index, is a good lumbar disease specific test, used commonly in patient followup after lumbar spine surgery [25, 26]. The newly developed ALSQ will be useful to give to patients preoperatively and postoperatively to assess for areas of improvement as well individual patient concern. The results of the ALSQ demonstrate that one-third of patients have a mild level of pain at their anterior incision. They have a low rate of new bowel or bladder complaints. The patient with retrograde ejaculation was discovered by his response on the ALSQ, not through chart review, and this demonstrates the ability for the questionnaire to provide a forum for surgeons and patients to discuss sensitive issues.

When evaluating risk factors for worse outcome, after anterior lumbar surgery using the ALSQ, smokers demonstrated the highest rate of worse patient response than their nonsmoking counterparts. This occurred on 13 questions. This is similar to many studies that show that patients who smoke have worse clinical outcome scores [27]. An additional risk factor for worse patient outcome is worker's compensation patients who had worse response on 8 questions, which has been shown in a recent study in posterolateral fusion to be an indicator for worse outcome on ODI and SF-36 [28].

A few anatomic risk factors for worse outcomes after anterior lumbar surgery were determined from this study. The increased rate of bulging or asymmetry that is associated with the L3/4 level may be related to an anatomic finding at that level such as anterior abdominal wall innervation usually overlying that area. Additionally, a significant association was found between sexual dysfunction and the fusion levels of L4-5 or L4-S1. The fusion level of L5-S1 has been previously shown as a higher risk for sexual dysfunction due to the presacral superior hypogastric plexus [14, 15, 18, 20, 29, 30]. Patients with isolated fusions at L5/S1 did not have a higher rate of sexual dysfunction and thus the L4-5 level should be considered at risk for sexual dysfunction.

## 5. Conclusions

Our newly proposed Anterior Lumbar Surgery Questionnaire directly assesses issues specific to patients undergoing anterior lumbar surgery. At 3 years of followup, the revision rate for anterior wound complications was only 1.6%, the overall complication rate was 10.7%, and there was one male patient (2.9%) with retrograde ejaculation. BMP use did not correlate with retrograde ejaculation or other sexual dysfunctions, and smoking was the risk factor associated with the highest rate of poor responses. Our survey was significantly associated with similar response on the ODI and EuroQol for similar questions types. The results of this survey demonstrate anterior specific considerations before attempting surgery. A patient must be informed of the potential side effects specific to the anterior approach such as incisional location, scarring, hernia, and pain, as well as potential bowel, bladder, and sexual function changes.

## Figures and Tables

**Table 1:** Patient demographics.

Age at surgery	49.2 years
Gender	
Women	47%
Men	53%
BMI	29.1
Smokers	17%
Worker's compensation	16%
Primary diagnosis	
DDD	74% (43)
Spondylolisthesis	26% (15)
Number of fusion levels^*^	
1	28
2	24
3	3
Specific levels	
L3/4 or L3-L5	7
L4/5 or L4-S1	28
L5/S1	23
Anterior only	8
Anterior-posterior	50
Posterior approach	
Percutaneous	40
Open	18

^*^3 patients not included due to the presence of lumbar disc replacements.

**Table 2:** Intraoperative patient characteristics.

Interbody device	
FRA	52.8%
Polyetheretherketone (PEEK)	26.4%
Metal	15.1%
TDR	5.7%
BMP used	
Yes	50%
No	50%
Anterior approach specific complications	
None	89.7%
Vascular complication	6.7%
Incisional hernia	1.7%
Retrograde ejaculation	3.2%
Anterior wound revisions	
None	98.3%
Abdominal hernia repair	1.7%

**Table 3:** Changes in HRQOL measures.

	Before op	Change	After op	*P* value
EuroQol	0.41	0.28	0.66	0.0001
ODI	58.79	34.94	31.99	0.0001

**Table 4:** Comparison of specific questions on the anterior lumbar surgery survey to EQ-5D and ODI questions.

	ODI pain	EQ-5D pain/discomfort	ODI personal care	EQ-5D self-care	ODI walk/sit/stand/travel	EQ-5D usual activities	ODI social life	ODI sex life	EQ-5D anxiety/depression
Anterior incision pain	0.05	0.011	>0.05	>0.05	>0.05	>0.05	>0.05	>0.05	>0.05
Anterior incision pain limits ADLS	>0.05	>0.05	0.0001	0.003	0.0001/0.012/0.015/0.0001	0.002	>0.05	>0.05	>0.05
Normal sex life	0.0001	0.0001	>0.05	>0.05	>0.05	>0.05	0.0001	0.0001	0.0001
Change in erection	0.024	>0.05	>0.05	>0.05	>0.05	>0.05	>0.05	>0.05	0.019
Maintain erection	0.008	>0.05	>0.05	>0.05	>0.05	>0.05	>0.05	>0.05	0.018
Ejaculate/maintain moisture	>0.05	>0.05	>0.05	>0.05	>0.05	>0.05	>0.05	0.014	0.011
Achieve orgasm	>0.05	>0.05	>0.05	>0.05	>0.05	>0.05	0.046	0.046	0.021
Change in genital sensation	0.011	0.002	>0.05	>0.05	>0.05	>0.05	0.004	0.017	0.003

## Appendix

### A. Anterior Lumbar Surgery Questionnaire (with Patient Response Rates)

#### A.1. Pain (only with regard to the anterior incision)

(a) Do you have pain in the area of your anterior incision?
  Yes, 35%/no, 65%
(b) If “yes,” please rate your pain:
(c) If “yes” to anterior incisional pain, how often do you have it?
  Everyday, 45%
  Frequently, but not daily (2-3 times a week), 27%
  Occasionally (once a week), 5%
  Rarely (once a month or less), 23%
(d) Since the first year following your surgery, has the pain/discomfort around your anterior incision changed?
  No change, 25%
  Improving, 30%
  Getting worse, 10%
  Never had any pain, 35%
(e) Do you have any persistent sensitivity or numbness along the anterior incision?
  Yes, sensitivity only, 7%
  Yes, numbness only, 20%
  Yes, both, 30%
  No, neither, 43%

#### A.2. Appearance

(a) Do you have any bulging or asymmetry near your anterior scar?
  Yes, 30%/no, 70%
(b) Please rate the appearance of your anterior incision and abdominal shape on a scale from 1 to 10.
  (1: very unsatisfactory, 10: very satisfactory)
(c) Has the shape of your abdomen changed since the first year after surgery?
  No change, 66%
  Improving, 21%
  Getting worse, 13%
(d) Have you had any further treatment specifically for or to your anterior incision?
  Yes, nonsurgical (medications, massage, etc.), 14%
  Yes, surgical repair, 2%
  No, 84%

#### A.3. Function

(a) Does your anterior incision on your abdominal area limit your ability to perform any activities comfortably (cleaning, cooking, self hygiene, exercise, etc.)?
  Yes, 19%/no, 81%
If “yes,” what activities?
(b) Compared with before your anterior abdominal surgery, how do you rate your current bowel function?
  Same, 81%
  Different-better, 5%
  Different-worse, 14%
(c) If your bowel function is worse now than before your spine surgery, your primary complaint will be
  Constipation, 72%
  Diarrhea, 21%
  Other, 7%
(d) Since your surgery, how often do you experience problems with your bowel habits?
  No change compared to before surgery, 66%
  Everyday, 8%
  Frequently, but not daily (2-3 times a week), 10%
  Occasionally (once a week or so), 7%
  Rarely (once a month), 10%
(e) Since your spine surgery, have you required medical evaluation or treatment for any new GI (Gastrointestinal/bowel) dysfunction?
  Yes, 13%/no, 87%
(f) Compared with before your anterior abdominal surgery, how do you rate your current urinary function?
  Same, 73%
  Different-better, 5%
  Different-worse, 22%
(g) If your urinary function is worse now than before your spine surgery, is your primary complaint:
  Urinary urgency, 33%
  Difficulty starting urination, 28%
  Urinary incontinence, 17%
  Other, 22%
(h) Since your surgery, how often do you experience problems with urination?
  No change compared to before surgery, 75%
  Everyday, 12%
  Frequently, but not daily (2-3 times a week), 8%
  Occasionally (once a week or so), 2%
  Rarely (once a month), 3%
(i) Since your spine surgery, have you required medical evaluation or treatment for any new urinary dysfunction?
  Yes, 14%/no, 86%
(j) Since your spine surgery how would you rate your current sexual function?
  My sex life is normal and causes no extra pain, 48%
  My sex life is normal but causes some extra pain, 18%
  My sex life is nearly normal but is very painful, 7%
  My sex life is severely restricted by pain, 8%
  My sex life is nearly absent because of pain, 12%
  Pain prevents any sex life at all, 7%

#### A.4. Gender Specific Sexual Function

*Men*

(a) Have you noticed any change in your ability to have an erection after the spine surgery?
  Yes, it is easier, 4%
  Yes, it is more difficult, 46%
  No change, 50%
(b) Have you noticed any change in your ability to maintain an erection after the spine surgery?
  Yes, it is easier, 4%
  Yes, it is more difficult, 57%
  No change, 39%
(c) Have you noticed any change in your ability to have an orgasm after the spine surgery?
  Yes, it is easier, 8%
  Yes, it is more difficult, 50%
  No change, 42%
(d) Have you noticed any change in your ability to ejaculate with orgasm since the spine surgery?
  Yes, my ejaculation is more, 4%
  Yes, my ejaculation is less, 36%
  Yes, I have no ejaculation with orgasm, 4%
  No change, 56%
(e) Have you noticed any change in sensation in your genital region after the operation?
  Yes, 34%/no, 66%
(f) Did you try but did not succeed in having children before the spine surgery?
  Yes, 0%/no, 100%
(g) After the spine surgery, have you tried, but not succeeded in having children?
  Yes, 0%/no, 100%
If you have answered “yes” to any question, please describe the changes that have taken place

*Gender Specific Sexual Function*

*Women*

(h) Have you noticed any changes in the moistness of your genital area with arousal during sex since your spine surgery?
  Yes, I am more moist than before, 3%
  Yes, I am less moist than before, 19%
  No change, 78%
(i) Have you noticed any change in your ability to have an orgasm after the spine surgery?
  Yes, it is easier, 7%
  Yes, it is more difficult, 16%
  No change, 77%
(j) Have you noticed any changes in sensation in your genital area after the spine surgery?
  Yes, 15%/no, 85%
(k) Did you try, but not succeed in having children before the spine surgery?
  Yes, 0%/no, 100%
(l) After the spine surgery, have you tried but did not succeed in having children?
  Yes, 0%/no, 100%
(m) Have you noticed any other changes in your genital area since the spine surgery?
  Yes, 0%/no, 100%

 If you have answered “yes” to any question, please describe the changes that have taken place.

Are there any other major problems or concerns regarding your belly or anterior incision that have not been covered?
